# Lessons learned from the initial sequencing of the pig genome: comparative analysis of an 8 Mb region of pig chromosome 17

**DOI:** 10.1186/gb-2007-8-8-r168

**Published:** 2007-08-17

**Authors:** Elizabeth A Hart, Mario Caccamo, Jennifer L Harrow, Sean J Humphray, James GR Gilbert, Steve Trevanion, Tim Hubbard, Jane Rogers, Max F Rothschild

**Affiliations:** 1 Wellcome Trust Sanger Institute, Wellcome Tust Genome Campus, Hinxton, Cambridge CB10 1SA, UK; 2 Centre for Integrated Animal Genomics, Kildee Hall, Iowa State University, Ames, IA 50011, USA

## Abstract

**Background:**

We describe here the sequencing, annotation and comparative analysis of an 8 Mb region of pig chromosome 17, which provides a useful test region to assess coverage and quality for the pig genome sequencing project. We report our findings comparing the annotation of draft sequence assembled at different depths of coverage.

**Results:**

Within this region we annotated 71 loci, of which 53 are orthologous to human known coding genes. When compared to the syntenic regions in human (20q13.13-q13.33) and mouse (chromosome 2, 167.5 Mb-178.3 Mb), this region was found to be highly conserved with respect to gene order. The most notable difference between the three species is the presence of a large expansion of zinc finger coding genes and pseudogenes on mouse chromosome 2 between *Edn3 *and *Phactr3 *that is absent from pig and human. All of our annotation has been made publicly available in the Vertebrate Genome Annotation browser, VEGA. We assessed the impact of coverage on sequence assembly across this region and found, as expected, that increased sequence depth resulted in fewer, longer contigs. One-third of our annotated loci could not be fully re-aligned back to the low coverage version of the sequence, principally because the transcripts are fragmented over several contigs.

**Conclusion:**

We have demonstrated the considerable advantages of sequencing at increased read depths and discuss the implications that lower coverage sequence may have on subsequent comparative and functional studies, particularly those involving complex loci such as *GNAS*.

## Abstract 2

The sequencing, annotation and comparative analysis of an 8Mb region of pig chromosome 17 allows the coverage and quality of the pig genome sequencing project to be assessed

## Background

The pig (*Sus scrofa*) occupies a unique position amongst mammalian species as a model organism of biomedical importance and commercial value worldwide. A member of the artiodactyls (cloven-hoofed mammals), it is evolutionarily distinct from the primates and rodents. At 2.7 Gb, the pig genome is similar in size to that of human and is composed of 18 autosomes, plus X and Y sex chromosomes. Extensive conservation exists between the pig and human genome sequence, making pig an important model for the study of human health and particularly for understanding complex traits such as obesity and cardiovascular disease. Alongside other recently sequenced mammalian species of biological significance, such as cow (sequenced to 7× coverage) and dog (sequenced to 7.5× coverage), the pig will be the next mammal to have its entire genome sequenced.

The Swine Genome Sequencing Consortium [1,2] has secured first phase funding from the USDA and many other institutions to achieve draft 4× sequence depth across the genome. The sequencing, being undertaken at the Wellcome Trust Sanger Institute, utilizes a bacterial artificial chromosome (BAC) by BAC strategy through a minimal tilepath provided by the integrated, highly contiguous, physical map of the pig genome [3,4]. Additional funding has been made available for increased sequencing on chromosomes 4, 7, 14 and the sequences of these chromosomes are now available from the PreENSEMBL website [5]. To test the usefulness of our approach to sequencing the pig genome and to obtain information for a quantitative trait locus (QTL) of interest, the *S. scrofa *physical map was used to identify a tilepath of 69 overlapping BACs across an 8 Mb region of SSC17 syntenic to human chromosome 20 (20q13.13-q13.33) and mouse chromosome 2 (167.5 Mb-178.3 Mb). For this study, the BACs were sequenced to a depth of 7.5× coverage and manually finished to High Throughput Genomic sequence (HTGS) Phase 3 standard. The high quality of the sequence enabled manual annotation to be performed using the same pipeline and standards as the GENCODE project [6].

Interest in pig chromosome 17 amongst researchers in the field of animal genomics has arisen following the identification of QTL on this chromosome that affect carcass composition and meat quality [7,8]. For medical scientists, the significance of this region lies in the presence of loci such as *PCK1 *and *MC3R*, which have been linked to diabetes and obesity in mammals [9,10]. Furthermore, loci in the vicinity of 20q13.2 have been found significantly amplified in a number of human breast and gastric cancers [11,12]. Manual annotation of genomic sequence remains the most reliable method of accurately defining the exon and intron boundaries of genes and identifying alternatively spliced variants. However, this process can only be performed on high quality, finished, genomic sequence. Automatic gene annotation can be performed on draft genomic sequence, but the overall outcome is dependent on a reliable assembly, which in turn relies on the overall depth of sequencing. We address the anomalies that can arise in lower quality sequence here by comparing the assembly and annotation of draft pig genomic sequence generated using three different depths of read coverage. Complex genomic regions, in particular, benefit from increased sequence depth to provide a reliable platform for meaningful annotation. On pig chromosome 17, one such region is the *GNAS *complex locus, which encodes the stimulatory G-protein α subunit, a key component of the signal transduction pathway that links interactions of receptor ligands with the activation of adenylyl cyclase. This locus is subject to a complex pattern of imprinting in human, pig and mouse, with transcripts expressed maternally, paternally and biallelically utilising alternative promoters and alternative splicing [13-17].

We compare our annotation of pig chromosome 17 with that for the syntenic regions of human chromosome 20 (20q13.13-q13.33) and mouse chromosome 2 (167.5 Mb-178.3 Mb). Both of these chromosomes have been manually annotated by the HAVANA team [18] at the Wellcome Trust Sanger Institute and the data are publicly available via the VEGA browser [19]. The identification of similarities and differences between species across syntenic regions provides a wealth of information that can relate to chromosome structure, evolution and gene function. In this instance, our annotation and comparative analysis of this region of pig chromosome 17 will be of value to researchers in the fields of agronomics, genomics and biomedical sciences.

## Results and discussion

### Sequence clone tilepath identification

The region reported is in two contigs of finished BACs linked by one overlapping, unfinished BAC [EMBL:CU207400]. A minimal BAC tilepath was selected by assessing shared fingerprint bands in the contact of positional information derived from BAC end sequence alignments to the human genome.

### Annotation of finished BAC sequence

**Figure 1:**
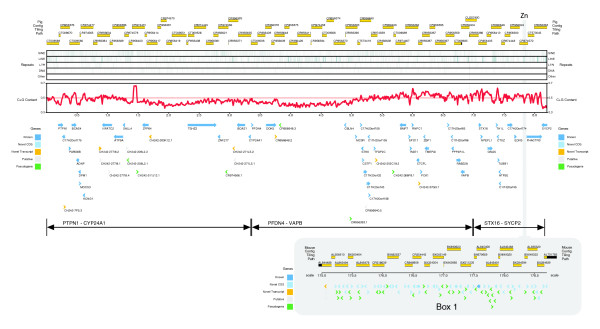
Feature map of the 8 Mb region of pig chromosome 17. Each locus is depicted according to type, orientation and position. The tiling path of the sequenced BACs is shown along the top. Below this, the distribution of repeats and C + G content is shown. Box 1 illustrates the zinc-finger locus expansion that has occurred in mouse between *EDN3 *and *PHACTR3*. The three regions described in the comparative analyses, *PTPN1-CYP24A1*, *PFDN4-VAPB *and *STX16-SYCP2*, are defined using double-headed arrows.

This 8 Mb region of pig chromosome 17 is represented by 69 BACs derived from either a CHORI-242 library or a Male Large White × Meishan F1 PigE BAC library. Within this region we identified and annotated 71 loci. Of these, we identified 53 loci that are orthologous to known human coding (CDS) genes, 7 novel transcripts, 5 putative novel transcripts and 6 processed pseudogenes. A brief description of each locus and its position within the region is summarized in Table 1 and a feature map of the overall region, including the BAC tiling path, is illustrated in Figure 1. All of these data are publicly available via the VEGA website. In Table 2, the number and type of loci within this region of pig chromosome 17 are compared to the syntenic regions of human and mouse. All three species contain very similar numbers of known coding genes but differ in the number of novel transcripts and putative loci. Specifically in mouse, the number of novel CDS and unprocessed pseudogene loci differ considerably from pig. We have divided this region of pig chromosome 17 into three sections to undertake comparisons with the syntenic regions of human chromosome 20 and mouse chromosome 2 in turn.

**Table 1:** List of manually annotated pig loci

Locus name	Locus description	Start coordinate	End coordinate
***PTPN1***	Tyrosine phosphatase 1B	192295	261948
***C17H20orf175***	Orthologue of human *C20orf175*	263263	296984
*CH242-7P5.3*	Novel transcript	291165	303697
***PARDB6***	Par-6 partitioning defective 6 homolog beta (*Caenorhabditis elegans*)	370305	389059
***BCAS4***	Breast carcinoma amplified sequence 4	414609	479471
***ADNP***	Activity-dependent neuroprotector	492879	525950
***DPM1***	Dolichly-phosphate mannosyltransferase polypeptide 1, catalytic subunit	529091	552289
***MOCS3***	Molybdenum cofactor synthesis 3	552563	554931
***KCNG1***	Potassium voltage-gated channel, subfamily G, member 1	600472	620055
*CH242-277I8.2*	Novel transcript	862318	889344
*CH242-277I8.1*	Putative novel transcript	891646	892957
***NFATC2***	Nuclear factor of activated T-cells	918501	1065177
*CH242-277I8.4*	Putative novel transcript	1034571	1035756
***ATP9A***	Atpase, class II, type 9A	1111110	1247324
***SALL4***	Sal-like 4 (*Drosophila*)	1257555	1278123
*CH242-209L2.2*	Putative novel transcript	1323104	1324011
*CH242-209L2.1*	Pseudogene similar to part of human protein regulator of cytokinesis 1 (*PRC1*)	1323229	1323658
*CH242-511J12.1*	Ribosomal protein L27a (*RPL27A*) pseuodgene	1496762	1497205
***ZFP64***	Zinc finger protein 64 homolog (mouse)	1577847	1666097
*CH242-300K12.1*	Novel transcript	1689884	1709546
***TSHZ2***	Teashirt family zinc finger 2	2317086	2773025
***ZNF217***	Zinc finger protein 217	2813463	2839285
*CR974566.1*	Thioltransferase (*GLRX1*) pseudogene	2937465	2937783
*CH242-271L5.2*	Novel transcript	3057211	3066632
*CH242-27L15.1*	Putative novel transcript	3077982	3079132
***BCAS1***	Breast carcinoma amplified sequence 1	3128687	3247224
***CYP24A1***	25-Hydroxyvitamin D3-24-hydroxylase	3318523	3339440
***PFDN4***	Prefoldin 4	3365055	3377364
***DOK5***	Docking protein 5	3600039	3751783
*CR956648.2*	Novel transcript	3756050	3764628
*CR956648.3*	Pseudogene similar to human *C11orf10*	3817744	3817975
***CBLN4***	Cerebellin precursor	4884433	4892814
*CR956393.1*	Ribosomal protein L27 (*RPL27*) pseudogene	4992534	4992878
***MC3R***	Melanocortin 3 receptor	5077795	5078875
***C17H20orf108***	Orthologue of human *C20orf108*	5151070	5162634
***STK6***	Serine/threonine kinase 6	5164100	5183118
***CSTF1***	Cleavage stimulation factor, 3' pre-RNA, subunit 1, 50 kda	5181641	5193333
***C17H20orf32***	Orthologue of human *C20orf32*	5200443	5240295
*CR956640.5*	Putative novel transcript	5224576	5225992
***C17H20orf43***	Orthologue of human *C20orf43*	5250245	5294040
***C17H20orf105***	Orthologue of human *C20orf105*	5271092	5277853
***C17H20orf106***	Orthologue of human *C20orf106*	5296497	5298326
***TFAP2C***	Transcription factor AP-2 gamma (activating enhancer binding protein 2 gamma)	5374025	5384555
*CH242-255C19.2*	Novel transcript	5409258	5410949
*CH242-266P8.1*	Ribosomal protein L27 (*RPL27*) pseudogene	5690286	5690695
***BMP7***	Bone morphogenetic protein 7 (osteogenic protein 1)	5794879	5886410
***SPO11***	SPO11 meiotic protein covalently bound to DSB-like (*Saccharomyces cerevisiae*)	5940695	5955823
***RAE1***	RAE1 RNA export 1 homolog (*Schizosaccharomyces pombe*)	5961819	5977901
***RNPC1***	RNA-binding region (RNP1, RRM) containing 1	5993104	6007945
***CTCFL***	CCCTC-binding factor (zinc finger protein-like)	6070196	6102129
*CH242-37G9.1*	Novel transcript	6114046	6116136
***PCK1***	Phosphoenolpyruvate carboxykinase 1 (soluble)	6140516	6146484
***ZBP1***	Z-DNA binding protein 1	6182270	6192447
***TMEPAI***	Transmembrane, prostate androgen induced RNA	6205610	6260081
***C17H20orf85***	Orthologue of human *C20orf85*	6580341	6590326
***C17H20orf86***	Orthologue of human *C20orf86*	6632471	6641977
***PPP4R1L***	Protein phosphatase 4, regulatory subunit 1-like	6644116	6665957
***RAB22A***	RAB22A, member RAS oncogene family	6721985	6779521
***VAPB***	VAMP (vesicle-associated membrane protein)-associated protein B and C	6800573	6850820
***STX16***	Syntaxin 16	6911191	6939474
***NPEPL1***	Aminopeptidase-like 1	6946539	6962106
***GNAS***	GNAS complex locus	7056486	7123907
***TH1L***	Th1-like (*Drosophila*)	7199960	7212384
***CTSZ***	Cathepsin Z	7212382	7220265
***TUBB1***	Tubulin, beta family 1	7232312	7239278
***ATP5E***	ATP synthase, H+ transporting, mitochondrial F1 complex, epsilon subunit	7241534	7245591
***C17H20orf45***	Orthologue of human *C20orf45*	7245938	7255973
***C17H20orf174***	Orthologue of human *C20orf174*	7384250	7452090
***EDN3***	Endothelin 3	7496706	7519565
***PHACTR3***	Phosphatase and actin regulator 3	7697213	7882258
***SYCP2***	Synaptonemal complex protein 2	7895310	7972154

The locus name, description and relative co-ordinates within the 8 Mb region are given. Locus names denoted in bold indicate that the locus is orthologous to a known human locus.

**Table 2:** Comparison of loci type and number in pig, human and mouse

Locus type	Pig	Human	Mouse
Known coding	53	54	52
Novel CDS	-	-	51
Novel transcript	7	15	22
Putative	5	24	12
Processed pseudogene	6	20	22
Unprocessed pseudogene	-	1	31
Expressed pseudogene	-	1	1
Total	**71**	**115**	**191**

### Comparative analysis: *PTPN1 *to *CYP24A1*

This region is well conserved between human, pig and mouse with respect to gene order. In human, this region (20q13.2) is of considerable interest because it is susceptible to amplification in a number of cancer lines, as shown by comparative genomic hybridization experiments [11,12,20]. In particular, *PTPN1*, *BCAS4*, *ZNF217 *and *CYP24A1 *have been found at increased copy numbers in human breast, ovarian, pancreatic and gastric cancer cell lines [12,21-23]. One noticeable difference between pig, mouse and human is the apparent absence of a *BCAS4 *counterpart in mouse. *BCAS4 *encodes a 203 amino acid protein of unknown function that shares homology with the cappuccino(*CNO*) locus in human, mouse and other mammalian species. We performed a BLASTP analysis to investigate whether a putative orthologue of *BCAS4 *could be found elsewhere in the mouse genome, using the predicted pig and human Bcas4 protein sequences to search ENSEMBL mouse (NCBI m36 assembly). However, the only homologous locus we identified in mouse was the *CNO *locus on chromosome 5. The relationship between Bcas4 and Cno homologues can be visualized using TREEFAM [24] [TREEFAM:TF326629]. In human, additional alternative splice variants of *BCAS4 *have been identified, with one potentially encoding a longer polypeptide of 211 amino acids. In human and mouse, five and seven novel transcripts or putative loci, respectively, lie between the *ZFP64 *and *TSHZ2 *loci. None of these appear to be conserved between the three species, and in pig only one novel transcript locus, *CH242-300K12.1*, was identified between *ZFP64 *and *TSHZ2*.

### Comparative analysis: *PFDN4 *to *VAPB*

Comparison of this region in pig, human and mouse reveals that it is highly conserved with respect to gene order and orientation. One notable difference between the three species in this region is the absence of porcine and murine counterparts of the human *C20orf107 *locus. In human, the *C20orf107 *locus lies immediately downstream of the *C20orf106 *locus. Both loci encode proteins of 171 amino acids and share 87% amino acid identity and 92% similarity. The function of these two proteins in human is unknown, although INTERPRO analysis predicts two transmembrane helices within these putative paralogues. The pig homologue of *C20orf106 *encodes a protein of 170 amino acids that shares 63% identity and 78% similarity with both human C20orf106 and C20orf107 proteins and contains these two putative transmembrane helices. To further investigate the presence of *C20orfl06 *and *C20orf107 *orthologues in other species, we compared this region across multiple organisms using ENSEMBL AlignSliceView [25]. Interestingly, it appears that the presence of both *C20orf106 *and *C20orf107 *loci is specific to primates: human, chimp and macaque all contain both *C20orf106 *and *C20orf107 *as neighboring loci whereas ENSEMBL non-primate species - for example, cow, rat and dog - appear to have only one or other of the two paralogues in the syntenic location. In the absence of additional species and a more detailed analysis it is not possible to draw definite conclusions regarding the evolutionary distribution of *C20orf106 *and *C20orf107*. However, these observations suggest that the absence of *C20orf107 *from this region in pig and mouse is not specific to these species.

### Comparative analysis: *STX16 *to *SYCP2*

The most striking difference between pig, human and mouse within this sub-region is the presence of a large cluster of zinc finger loci in mouse, between *Edn3 *and *Phactr3*, that is completely absent from pig and human. This mouse-specific expansion is over 3.2 Mb in length and contains one known coding gene, 51 genes with a novel CDS and 30 unprocessed pseudogenes, all predicted to contain C2H2 Zinc finger type and KRAB box domains. These motifs have been found to confer DNA binding ability and behave as transcriptional repressor domains in a number of proteins [26]. Given that the full extent of duplication within this region of the mouse genome is still being resolved, there is potential for the total number of loci to be even greater.

**Figure 2:**
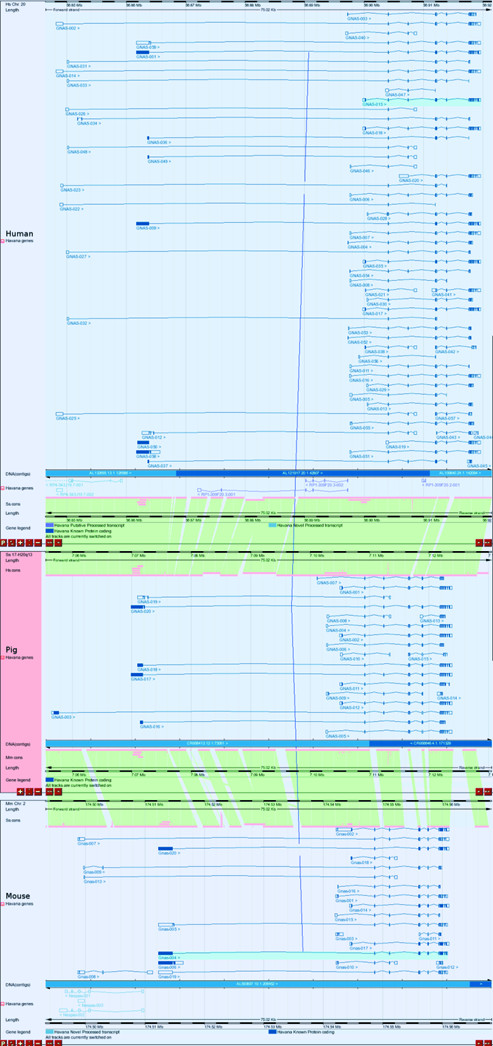
Comparison of *GNAS *transcripts in human, pig and mouse. A screenshot taken from VEGA Pig MultiContigView, comparing *GNAS *transcripts annotated in human (top panel), pig (middle panel) and mouse (bottom panel). The vertical blues lines joining loci in VEGA MultiContigView represent orthologous relationships between loci across species.

In contrast to the significant differences between pig and mouse between the *EDN3 *and *PHACTR3 *loci, the rest of this sub-region remains highly conserved across the three species, including the *GNAS *locus, one of the most complex loci to be found in mammalian genomes. A comparison of the *GNAS *transcripts annotated in pig, human and mouse can be viewed directly in VEGA using Pig MultiContigView [27], as is shown in Figure 2. To generate this simultaneous view of *GNAS *transcripts in all three species, pig *GNAS *should be viewed in VEGA ContigView. 'Homo_sapiens chromosome 20' should then be chosen from the 'View alongside' menu and 'Mus_musculus:2' added from the 'Comparative' drop-down menu. *GNAS *has been well studied in human and mouse and encodes four proteins - Gsα, Nesp Xlαs and Alex - that have been well-characterized in both species. Of these *GNAS *products, the most well-conserved are the alternatively spliced variants of Gsα, the alpha-stimulatory subunit of GTP-binding protein, which is biallelically expressed in human and mouse. The best known of these Gsα isoforms is 394 amino acids long in all three species. In pig and human these Gsα proteins are 100% identical with respect to primary structure, while the mouse orthologue differs by the substitution of just one amino acid. Paternally expressed, the large variant of G-protein α subunit known as Xlαs utilizes a large, upstream first exon compared to the Gsα variants [14,28]. The pig Xlαs homologue is predicted to be 1,005 amino acids long and shares 78% identity and 82% similarity with the human and 65% identity and 70% similarity with the mouse Xlαs proteins, which are 1,037 and 1,133 amino acids long, respectively. The capacity to encode the most unusual of the *GNAS *products, Alex, is also conserved in pig. Alex is translated in a different reading frame to Xlαs and has been described in rat and human [16,29]. The pig Alex protein is predicted to be 564 amino acids long while the human and mouse Alex proteins are 625 amino acids and 725 amino acids long, respectively. This difference in length is partly due to divergence within a proline-rich and leucine-rich stretch of amino acids that lie between residues 298 and 398 in porcine Alex. Alignment of these predicted pig, mouse and human Alex proteins reveals they are less conserved than the other *GNAS*-encoded proteins: pig Alex protein shares approximately 61% identity and 70% similarity with human Alex protein and 44% identity and 51% similarity with mouse Alex protein. Finally, expressed exclusively from maternal alleles in human and mouse, the *NESP55 *transcript encodes neuroendorine secretory protein 55. Pig Nesp55 shares 82% identity and 89% similarity with human Nesp55 (68% identity and 80% similarity with mouse Nesp55). At the mouse and human *GNAS *loci, maternally imprinted *NESP55 *antisense transcripts have been identified [30-32], unofficially known as *Nespas *and *SANG*, respectively. However, we have been unable to identify a pig *GNAS *antisense transcript. Pig has diverged sufficiently from human and mouse such that the exons of these antisense transcripts are not conserved. In human, *GNAS *appears to be the only locus that is imprinted within this region, 20q13.32: the two genes, *TH1 *and *CTSZ*, which lie downstream of *GNAS*, have been found to be biallelically expressed [33].

### Comparison of draft sequence assemblies

The manual annotation produced in this project is not only useful for comparative analyses but also can be used as a reference set to judge the influence of sequence coverage on gene annotation. For the purpose of this study, our 8 Mb region of pig chromosome 17 was sequenced to a depth of 7.5× coverage and manually finished to GenBank HTGS Phase 3 standard to produce sequence with a predicted error rate of less than 1 in 100,000 bases. However, the international pig genome sequencing project currently has funding to generate in the first phase of sequencing only draft sequence at 3-4× coverage overall (with the exception of chromosomes 4, 7 and 14, which will be sequenced to an improved draft using sequence targeted to close gaps). To assess the impact of sequencing coverage on contig size and gene integrity, we automatically assembled sequence reads obtained from 384-well plates of shotgun sequencing to represent differing amounts of coverage across the region: 2.5×, 5× and 7.5× (see Materials and methods for details).

**Figure 3:**
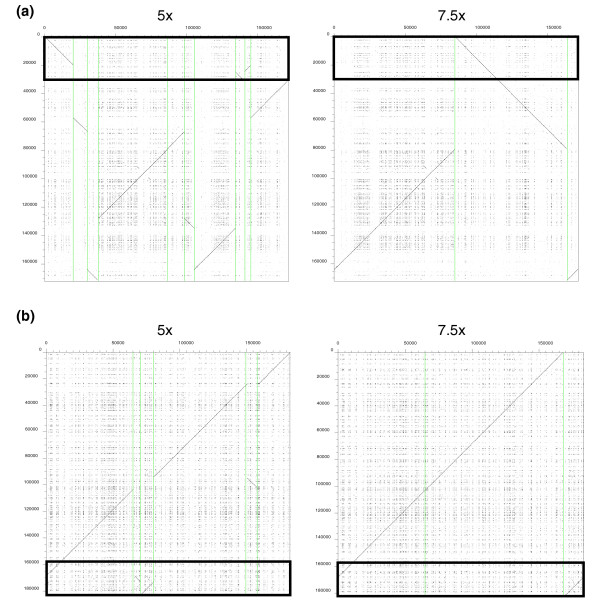
Comparison of 5× and 7.5× coverage assemblies. Dot-plots of finished BAC sequence against either 5× or 7.5× assembled sequence for BACS **(a) **CH242-247L10 and **(b) **CH242-155M9. Individual contigs, represented on the x-axis, are separated by vertical green lines. In (a) the black rectangle depicted on the graphs represents the *GNAS *downstream region. In (b) the black rectangle depicted on the graphs defines the vicinity of the pig *C20orf106 *locus.

We chose to count only contigs greater than 2 kb in our analysis, thus excluding short bacterial contaminants and single pass reads. When we assembled reads at a depth of 2.5× coverage, the mean number of contigs obtained per clone was 27 and the average total contig length was 138 kb. If coverage is increased to 5×, the mean number of contigs obtained per clone decreases to 13 and the average total contig length increases to 179 kb. When we increased the level of coverage further to 7.5× the mean number of contigs obtained per clone is reduced to 5 and the average total contig length achieved is 184 kb. Therefore, increasing the read coverage for each BAC clone results in fewer, longer contigs per clone. These results are illustrated in Figure 3, where the difference in contig number obtained after automatic assembly of reads at a level of either 5× and 7.5× coverage is represented using dot-plots for two different BAC clones: CH242-247L10 [EMBL:CR956646] and CH242-155M9 [EMBL:CR956640]. CH242-247L10 contains the 3' end of the *GNAS *complex locus and the downstream *TH1L*, *CTSZ*, *TUBB*, *ATP5E*, *C17H20orf45 *loci. At a level of 5× coverage, CH242-247L10 is assembled into 10 contigs longer than 2 kb, with the 50 kb region containing the 3' end of *GNAS *and its immediate downstream region (defined by a black rectangle) dispersed over 4 contigs. However, increasing the level of coverage to 7.5× reduced the total number of contigs longer than 2 kb to three, such that the *GNAS *downstream region is now contained within a single contig. A manual finishing step is still required to link these 3 contigs, but the assembly is much improved in comparison. In Figure 3b, CH242-155M9 contains the pig *C20orf106 *gene. As mentioned previously, pig lacks the paralogous locus, *C20orf107*, which lies immediately downstream of *C20orf106 *in human. At a depth of 5× coverage, CH242-155M9 is assembled into six contigs longer than 2 kb, with the region immediately downstream of *C20orf106 *(defined by a black rectangle) divided between three of these contigs. Using this assembly, it may not be easily ascertained whether the *C20orf107 *gene is absent in pig or falls within a gap in the assembly. Increasing the coverage to 7.5× decreases the total number of contigs to three (again, a manual finishing step would be required to link these three contigs) and we can be more confident that the *C20orf107 *locus is absent in pig and does not simply fall within a gap in the assembly.

Using EXONERATE [34] in conjunction with a splice-aware model, we investigated whether our manual annotation performed on the finished BACs could be aligned back to the 2.5×, 5× and 7.5× assemblies. In total, 71 loci were annotated within the finished BACs. For each of these genes we selected the longest transcript and discarded any that spanned multiple finished clones, leaving us with 58 transcripts, which we attempted to align back to the 2.5×, 5× and 7.5× assemblies. We counted only transcripts that could be fully re-aligned along their entire length. From the pool of 58 annotated transcripts we were able to fully re-align 54 to our 7.5× assembly, 39 to our 5× assembly and just 10 to our 2.5× assembly. This means that 33% of our annotated transcripts could not be fully re-aligned to the 5× assembly. Where re-alignment was unsuccessful, the most common reason was that the transcript spanned multiple contigs. In other instances, however, re-alignment failure was linked to mis-assemblies and low quality regions.

These results indicate that the impact of low-coverage sequencing on the structure of the assembly is considerable. Reducing the number of sequence reads from a depth of 7.5× to 5× and 2.5× increases the number of contigs within the assembly, decreases the total length of contigs and is likely to introduce errors in sequence organization due to the presence of gaps in sequence coverage. As a result, annotation of gene loci will be less precise and large genes are likely to be incomplete or artificially re-arranged.

## Conclusion

The generation and manual annotation of this 8 Mb region of pig chromosome 17 will provide a useful resource for researchers in the field of pig genomics, as well as scientists with a more general interest in mammalian comparative genomics. Importantly, we have also shown that increasing the sequence depth across this region of the pig genome has several material advantages with respect to coverage and quality.

We have identified 71 loci that lie between *PTPN1 *at the centromeric end of pig chromosome 17 and *SYPC2 *at the telomeric end. Comparison of this region with the 9.38 Mb and 10.8 Mb syntenic regions of human chromosome 20 and mouse chromosome 2, respectively, has revealed both striking similarities and differences between the three species. The most significant difference between pig, human and mouse is the presence of a 3.2 Mb expansion of zinc finger loci in mouse, absent in human and pig, which has occurred between *Edn3 *and *Phactr3 *andcould represent an event of evolutionary significance in the mouse lineage. Additional differences between the three species include the existence of *C20orf107 *in human that is absent from pig and mouse and the absence of the *BCAS4 *locus from mouse that is conserved in human and pig. We detected 12 transcribed non-coding loci specific to pig that may warrant further investigation. Eight of these lay between *PTPN1 *and *CYP24A1*, a region of interest subject to amplification in human cancer cell lines and associated with complex traits such as type 2 diabetes [35,36]. Furthermore, our annotation of the porcine orthologue of *GNAS *will contribute towards the characterization of this enigmatic complex locus. The predicted primary structures of the four putative pig *GNAS *products - Gsα, Xlαs, Alex and Nesp - are comparable to their counterparts in human and mouse. Interestingly, imprinted regions on other pig chromosomes have been linked to a range of QTLs [37], which suggests the region encompassing the pig *GNAS *locus is worthy of further analysis.

In addition to providing locus information within the confines of the sequence, we have used this test region of pig chromosome 17 to demonstrate the value of genome sequencing at increased levels of coverage. The advent of large-scale sequencing projects in the last two decades has been accompanied by the formulation of mathematical models to quantitatively determine the strategic design of such projects. The models proposed by Lander and Waterman [38], which extended the earlier theories of Clarke and Carbon [39], have provided theoretical guidelines for standard fingerprint mapping and shotgun sequencing projects and have been developed by others [40,41] as the nature and scale of sequencing projects has evolved. These algorithms continue to be relevant, particularly to assess the design, quality and value of new sequencing technologies and their applications to projects such as re-sequencing [42,43], which themselves will bring new challenges to the field. In this study, we have not set out to perform a detailed quantitative investigation into the effect of sequence depth on sequence assembly. However, we have taken advantage of this test region of the pig genome to illustrate the impact of read coverage on the structure and contiguity of the pig genome assembly and, importantly, annotation. We have shown that increasing sequence coverage from 5× (which is above the overall target depth of the pig genome) to 7.5× greatly improves the assembly of sequence reads into contigs. Specifically, it results in fewer and longer contigs, which improves the reliability of the genome assembly overall. A high degree of confidence in the fidelity of the genome assembly is advantageous in complex regions - for example, *GNAS *- that may contain non-coding regulatory sequences. It is preferable that such regions are kept as intact as possible, but our analysis showed the region just downstream of the *GNAS *locus to be fragmented over four contigs using the 5× assembly. Assembly errors that occur in intergenic regions may not be immediately obvious, but can have implications for subsequent analyses of non-coding regions. Using the *C20orf106*/*C20orf107 *loci in human as a second example, we showed that 5× coverage is insufficient to determine with confidence whether a pig orthologue of *C20orf107 *is absent from the pig lineage or simply falls within a gap in our assembly. Clearly, it is important to eliminate doubts such as these for meaningful comparative analyses. Genome annotation, whether automated or manual, is highly dependent on the integrity of the genome assembly. While reduction of errors at the base level is pertinent to improving the quality of shotgun sequence [44], our pilot study has focused on the impact of sequence structure on the quality of the final product. In particular, we assessed the effect of read coverage on genome annotation. We found that we were unable to fully re-align one-third of our annotated transcripts back to the 5× assembly, indicating that multiple contigs, gaps and assembly errors caused by low coverage sequencing significantly affect the quality of genome annotation. The value of a genome is dependent on the quality of its annotation, which makes sequencing coverage an important consideration in project design. There is no doubt that the 3-4× sequencing of the pig genome will provide researchers with another extremely valuable layer of information for mammalian comparative studies. However, the additional advantages that could be gained by additional investment should not be underestimated. Improving the level of sequencing coverage will undoubtedly provide a better platform for automated annotation and downstream analyses. Given the importance of pig as an agricultural species and a biomedical model, greater advances in many aspects of porcine and mammalian science might be made if further funding was made available to improve the overall coverage of the entire pig genome.

## Materials and methods

### Mapping and sequencing

A physical map of the porcine genome was constructed using the fingerprints and end sequences generated from over 264,000 BACs from 4 BAC libraries and ordering information derived from pig radiation hybrid markers and sequence homology to the human genome. The current assembly contains just 172 contigs and covers >98% of the genome.

Sequence clones were sub-cloned into 4-6 kb inserts in pUC 19 and sequenced to up to 8-fold depth with Applied Biosystems (Foster City, CA, USA) Big Dye v3 chemistry. Sequence reads were assembled using PHRAP. Assembled clones were improved by one round of primer walking to extend sequence contigs and close gaps before the clones were examined and final gap closure and checking procedures were carried out. The integrity of the finished clones was assessed by reference to three restriction enzyme digests compared to virtual digestions performed on the sequence assembly before sequence accessions were declared finished and entered into EMBL/GenBank HTGS Phase 3.

### Sequence annotation

Manual annotation was performed on the pig genomic sequence by the Wellcome Trust Sanger Institute Havana team as follows: The finished porcine sequence was analyzed using an automatic ENSEMBL pipeline [45] with modifications to aid the manual curation process. The G + C content of each clone sequence was analyzed and putative CpG islands were marked. Interspersed repeats were detected using RepeatMasker using the mammalian library along with porcine-specific repeats submitted to EMBL/NCBI/DDBJ and simple repeats using Tandem Repeats Finder [46]. The combination of the two repeat types was used to mask the sequence. The masked sequence was searched against vertebrate cDNAs and expressed sequence tags (ESTs) using WU-BLASTN and matches were cleaned up using EST2_GENOME. A protein database combining non-redundant data from SwissProt and TrEMBL was searched using WU-BLASTX. *Ab initio *gene structures were predicted using FGENESH and GENSCAN. Predicted gene structures were manually annotated according to GENCODE standards [6]. The gene categories are described on the VEGA website [19]: 'Known' genes are identical to known pig cDNAs or are orthologous to known human loci; 'Novel CDS' loci have an open reading frame (ORF), are identical to spliced ESTs or have some similarity to other genes and proteins; 'Novel transcript' is similar to novel CDS but no ORF can be determined unambiguously; 'Putative' genes are identical to spliced pig ESTs but do not contain an ORF; and 'Pseudogenes' are non-functional copies of known or novel loci.

### Comparison of draft sequence assemblies

We calculated the three depths of coverage (2.5×, 5× and 7.5×) that were compared across this particular region as follows. We predicted an insert size of 173 kb for our BAC libraries and the average read length achieved during sequencing was 713 base pairs. Therefore, for this region, approximately 240 sequencing reads represent a depth of 1× coverage. For each BAC, approximately 600 passed reads were obtained from a 384-well plate after quality checking. Thus, one plate of 600 passed reads represents approximately 2.5× coverage for that clone; two plates constitute around 1,200 passed reads and is equivalent to up to 5× coverage; three plates constitute approximately 1,800 passed reads and is equivalent to up to 7.5× coverage. Using PHRAP, we automatically re-assembled 62 BAC clones from the 8 Mb region using one, two and three plates of passed reads to obtain the 2.5×, 5× and 7.5× assemblies, respectively. Assembled contigs that were shorter than 2 kb were discarded. The resulting assemblies for each clone were compared to each other directly with respect to contig number and length. Our manually annotated loci were re-aligned to each of the three assemblies using EXONERATE [34] in conjunction with a splice-aware model to avoid spurious hits. For each annotated locus we selected the longest transcript (where alternative variants had been annotated) but discarded transcripts that spanned multiple finished clones. Thus, we attempted to re-align a total of 58 transcripts to our 2.5×, 5× and 7.5× assemblies. Only transcripts that could be re-aligned entirely back to the assembly across their full length were counted as being successfully re-aligned. All of the sequence traces from this project have been deposited in the trace repository and are available from the ENSEMBL trace server [47].

## Abbreviations

BAC, bacterial artificial chromosome; EST, expressed sequence tag; QTL = quantitative trait locus.

## Authors' contributions

Manual annotation of finished pig BACs and subsequent comparative analysis was undertaken by EA Hart. The comparison of draft pig sequence assemblies was performed by M Caccamo.
